# Enhanced strength and ductility in a friction stir processing engineered dual phase high entropy alloy

**DOI:** 10.1038/s41598-017-16509-9

**Published:** 2017-11-23

**Authors:** S. S. Nene, K. Liu, M. Frank, R. S. Mishra, R. E. Brennan, K. C. Cho, Z. Li, D. Raabe

**Affiliations:** 1 Center for Friction Stir Processing, Department of Materials Science and Engineering, University of North Texas, Denton, Texas 76203 USA; 2 Weapons and Materials Research Directorate, U.S. Army Research Laboratory, Aberdeen Proving Grounds, MD, 21005 USA; 3 Max-Planck-Institut für Eisenforschung, Max-Planck-Str. 1, 40237 Düsseldorf, Germany

## Abstract

The potential of high-entropy alloys (HEAs) to exhibit an extraordinary combination of properties by shifting the compositional regime from the corners towards the centers of phase diagrams has led to worldwide attention by material scientists. Here we present a strong and ductile non-equiatomic HEA obtained after friction stir processing (FSP). A transformation-induced plasticity (TRIP) assisted HEA with composition Fe_50_Mn_30_Co_10_Cr_10_ (at.%) was severely deformed by FSP and evaluated for its microstructure-mechanical property relationship. The FSP-engineered microstructure of the TRIP HEA exhibited a substantially smaller grain size, and optimized fractions of face-centered cubic (f.c.c., γ) and hexagonal close-packed (h.c.p., ε) phases, as compared to the as-homogenized reference material. This results in synergistic strengthening via TRIP, grain boundary strengthening, and effective strain partitioning between the γ and ε phases during deformation, thus leading to enhanced strength and ductility of the TRIP-assisted dual-phase HEA engineered via FSP.

## Introduction

High-entropy alloys (HEAs) represent a special class of materials that were orginaly designed to obtain a single-phase massive solid solution devoid of any secondary phases^1^. The approach provides high solid solution strengthening and may suppress formation of brittle intermetallic phases^1,2^. Significant efforts have been devoted to develop new HEAs for overcoming the strength-ductility trade-off inherent in most conventional materials. A prominent example is an equiatomic HEA with composition Fe_20_Mn_20_Ni_20_Co_20_Cr_20_, which showed excellent strength, ductility, and fracture toughness at cryogenic and room temperatures^2^. However, the limitation of the equi-atomic HEA approach is that it represents a single point within a huge compositional phase space. Thus, a number of non-equiatomic HEAs such as Al_1.5_CoCr_0.5_FeNi_0.5_ and different variants of the FeMnNiCoCr system were introduced, showing in part promising property profiles^3–6^.

HEAs have shown a strong potential in tuning their primary deformation mechanisms by adjusting their chemical composition and by engineeering the microstructure via processing. Recent work by Li *et al*.^6^ demonstrates tunability of deformation mechanisms such as dislocation slip, twin induced plasticity (TWIP) and trnasformation induced plasticity (TRIP) by varying the Mn content from 45 to 30 at % in the Fe-Mn-Co-Cr system. This led to the developement of another class of HEAs known as dual phase Fe_50_Mn_30_Co_10_Cr_10_ HEA which utlizes the TRIP effect as the primary strain accomodation mechanism during plastic deformation. In this material a simultaneous increase in strength and ductility was obtained due to the engineered fraction and thermodynamic stability of the face-centered cubic (f.c.c., γ) and hexagonal close-packed (h.c.p., ε) phases in the microstructure through well designed composition and thermomechanical processing^3,4,6^. While such microstructures and their effects associated with conventional thermomechanical processing on the deformation behavior in HEAs, including TRIP HEAs, have been investigated in detail, studies on severe plastic deformation, such as equal channel angular pressing (ECAP), high-pressure torsion (HPT) and friction stir processing (FSP), of HEAs and their effects on the mechanical properties^7–9^ have been limited so far. Among these techniques, FSP is the most industrially feasible process for bulk products and has applicability for solid state joining. Earlier work by Kumar *et al*.^9^ showed that FSP of Al_0.1_CoCrFeNi HEA resulted in substantial improvement of strength and ductility when compared with the as-cast condition, due to enhanced grain refinement and a greater fraction of high angle grain boundaries. The difference between FSP and ECAP/HPT lies in the pathway of microstructural refinement. The fine grain size obtained from FSP results from dynamic recrystallization and limiting subsequent grain growth^10^. Grain refinement during ECAP and HPT occurs by deformation-driven grain fragmentation and patterned dislocation storage leading to a high fraction of low angle grain boundaries^11,12^.

Li *et al*.^3,4^ had shown that the strength-ductility profile of this material depends not only on grain size but also on the fraction of the h.c.p. ε phase and the density of stacking faults^4^. FSP leads to a highly transient microstructure type, as the material is deformed at a high temperature and individual grains experience different stages of straining. Here we used FSP to engineer the microstructure of the TRIP Fe_50_Mn_30_Co_10_Cr_10_ HEA along two strands of motivation. The first is that grain size reduction enhances the stability of the f.c.c. γ-phase grains against deformation-driven transformation. This effect should lead to a more uniform distribution of the f.c.c. γ → h.c.p. ε transformation zones, thereby maximizing the dispersion and hence the TRIP effect^3^. Second, joinability of any advanced novel alloy is an essential precondition for using it in engineering applications. FSP results provide a first overview of the alloy’s suitability for friction stir welding.

## Results and Discussion

### Microstructure evolution after FSP

**Figure 1:**
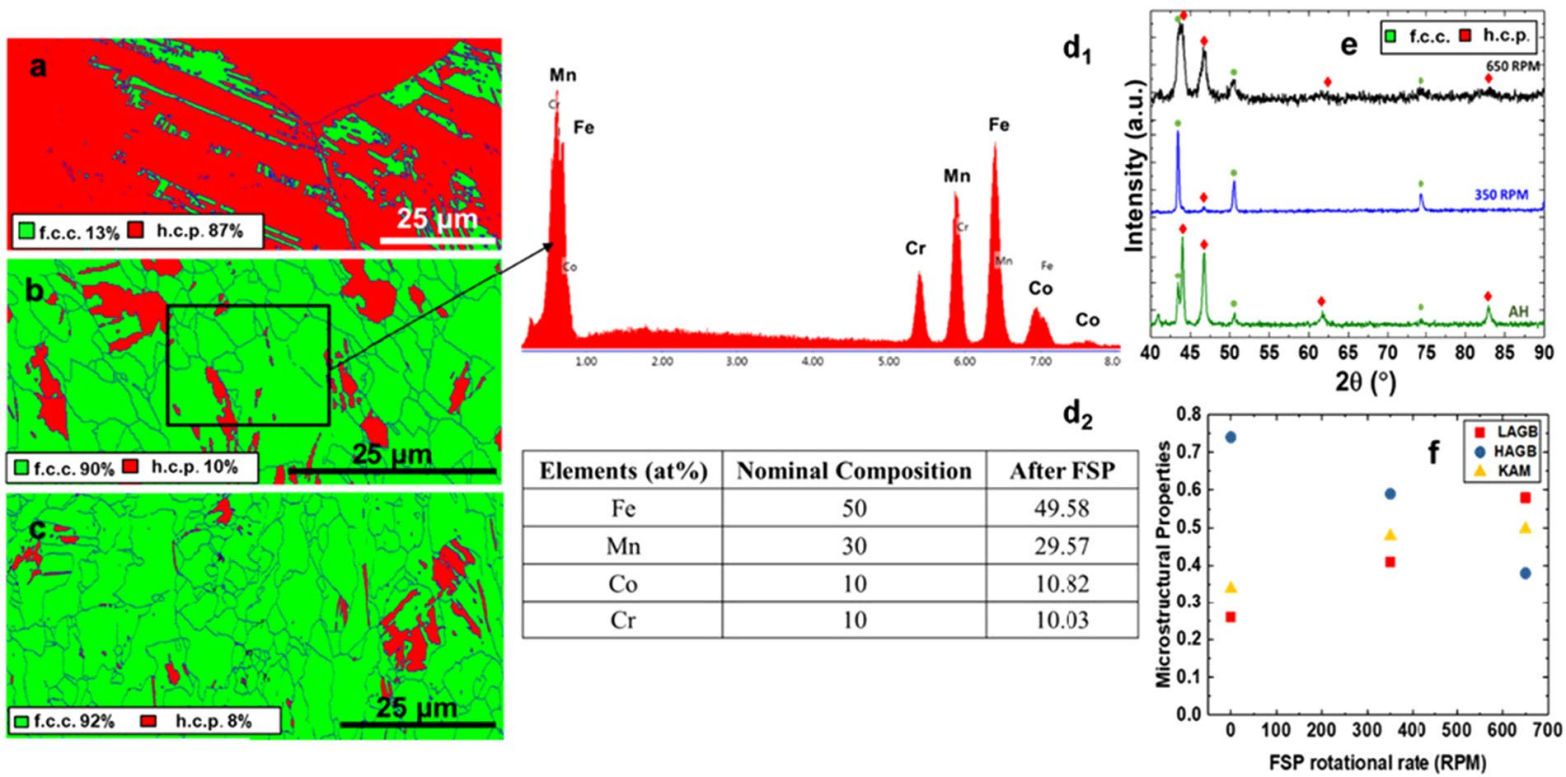
(**a**–**c**) Phase maps for the material in the as-homogenized condition; after additional 350, and after 650 RPM processing, (**d**_**1**_–**d**_**2**_) corresponding EDS results for 350 RPM processed sample, (**e**) corresponding XRD results for the as-homogenized and FSP samples, (**f**) microstructural properties including the fraction of high angle grain boundaries, low angle grain boundaries, and kernel average misorientation values after FSP. AH: as-homogenized; FSP: friction stir processing; RPM: rotations per minute; LAGB: low angle grain boundary; HAGB: high angle grain boundary.

Figure 1a–c show electron back scattered diffraction (EBSD) maps for the as-homogenized and 350 and 650 rotations per minute (RPM) treated FSP samples, highlighting the drastic reduction in average grain size from ~100 µm to 6.5 and 5.2 µm, respectively. Since FSP is a high temperature, severe plastic deformation process, it also changed the fraction of f.c.c. γ and h.c.p. ε-phases in the microstructure while maintaining the chemical homogeneity. Fig. 1d_1_,d_2_ display the EDS analysis results for a 350 RPM specimen showing average compositions of the constituent elements of the dual phase (DP)-HEA suggesting homogeneity in the microstructure. As discussed by Palanivel *et al*.^10^, FSP leads to a homogenization of the microstructure and its local composition owing to the enhanced shear-related deformation-driven transport and mixing of the elements. XRD analysis (Fig. 1e) shows the phase changes that have occurred with FSP, as depicted by the variation in peak intensities for f.c.c. γ and h.c.p. ε-phases as a function of FSP tool rotation rates. FSP led to a significantly higher fraction of f.c.c. γ-phase in the microstructure of the TRIP HEA irrespective of the tool rotational rates as seen in Fig. 1b–d in comparison with the as-homogenized reference condition (Fig. 1a).

A detailed analysis of the EBSD data (Fig. 1f) revealed that the fraction of high-angle grain boundaries (HAGBs), as highlighted by blue lines, decreased in comparison to the as-homogenized material as a result of severe plastic deformation during FSP. This fractional drop of HAGBs was also supported by the higher kernel average misorientation (KAM) values for the 350 and 650 RPM processed samples as compared to the as-homogenized microstructure. However, the KAM values were less than 1.2 irrespective of the processing condition, which also indicated the occurrence of dynamic recrystallization upon FSP. Moreover, most of the new grains were twin-free, and h.c.p ε was present in the form of plates within the f.c.c. grains as shown in Fig. 1b,c.

Li *et al*.^3,4^ reported ~32% of h.c.p. phase in the microstructure with an average f.c.c. γ grain size of 4.5 μm upon cold-rolling and subsequent annealing at a furnace temperature of 900 °C for 3 min. After FSP we find that the same alloy showed ~8 and ~10% of h.c.p. at similar grain sizes of ~5.2 and 6.5 μm, respectively. The TRIP effect includes the conversion of f.c.c. γ-phase to h.c.p. ε-phase under deformation, and thus a higher starting fraction of f.c.c. γ-phase should lead to a higher level of TRIP effect, provided that the thermodynamic stability of the f.c.c. γ-phase is similar in both cases. Therefore, FSP provided an expedient route for obtaining fine-grained TRIP HEAs with higher f.c.c. γ-phase fraction, as compared to conventional thermomechanical processing, which required multiple processing steps.

### Stress-strain behavior

**Figure 2:**
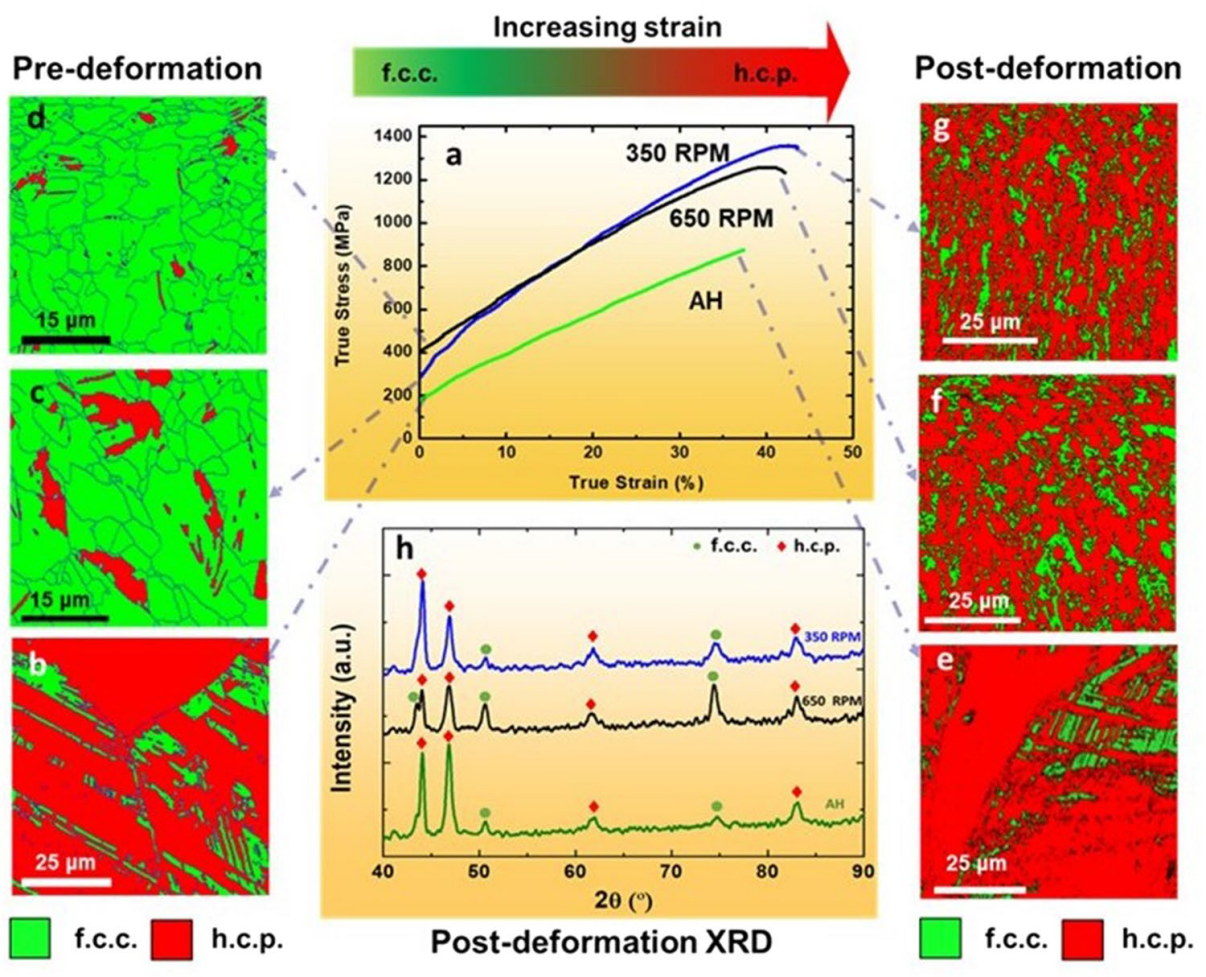
(**a**) True stress-true strain curves for the as-homogenized and FSP samples deformed at room temperature at an initial strain rate of 10^−3^ s^−1^, (**b**–**d**) EBSD maps showing f.c.c. γ- and h.c.p. ε-phase fractions prior to tensile deformation and, (**e**–**h**) EBSD maps and corresponding XRD patterns showing f.c.c. γ- and h.c.p. ε-phase fractions after tensile deformation. AH: as-homogenized; RPM: rotations per minute.

Figure 2a–g present the true stress-true strain curves for the 350 and 650 RPM treated samples, along with the EBSD phase maps before and after tensile deformation, as compared to the as-homogenized reference material. Since the material in as-homogenized state had coarser grains and a higher ε-phase content (Fig. 2b), it also exhibited lower strain hardening response, reaching a maximum true stress of 800 MPa with uniform elongation of 35%. The as-homogenized material experienced a limited TRIP effect, as indicated by the similar color codes in the EBSD phase maps (Fig. 2b,e), and the XRD data obtained before and after tensile deformation (Fig. 2h). The FSP treatment led to significant improvement in the tensile mechanical properties of the alloy, i.e. maximum true stress values of 1400 MPa and 1200 MPa at uniform elongations of almost 45% and 42% were obtained for the samples with tool rotational rates of 350 and 650 RPM, respectively (Fig. 2a). This enhanced combination of strength and ductility was partly attributed to ~90% f.c.c. γ-phase, as shown in Fig. 2c,d prior to deformation, and the associated high TRIP-related strain hardening during deformation.

The change in color from green to red shown by the EBSD phase maps (Figs 2c,d,f,g) and majority h.c.p. ε-phase peaks shown by XRD (Fig. 2h) after failure of the FSP tensile samples confirmed the deformation-induced phase transformation from f.c.c. γ- into the h.c.p. ε-phase. Tensile deformation resulted in transformation of ~90% of the starting γ-phase to almost 79% and 75% h.c.p. ε-phase for the 350 and 650 RPM conditions, respectively.

The values of true ultimate stress and ductility for the 350 RPM treated sample appear similar to the highest values reported by Li *et al*.^3,4^ after cold-rolling and 3 min annealing. Moreover, the properties of the single phase equi-atomic Fe_20_Mn_20_Co_20_Cr_20_Ni_20_ alloy appeared to be significantly inferior to those observed for the FSP alloys having similar grain sizes^11^. The work hardening response of the 350 RPM treated and as-homogenized reference material after tensile deformation confirmed that FSP produced a pronounced increase in work hardening. Regarding the yield strength (YS) values of the alloy, significant improvement of the 350 RPM treated sample led to a value of 298 MPa compared to a value of 198 MPa for the as-homogenized sample. The 650 RPM treated sample showed ~200 MPa increase in YS (Fig. 2a) compared to the as-homogenized condition. These improved values for the FSP specimens were related to more homogeneous deformation because of finer grains in comparison with the as-homogenized material.

### Deformation mechanisms after FSP

**Figure 3:**
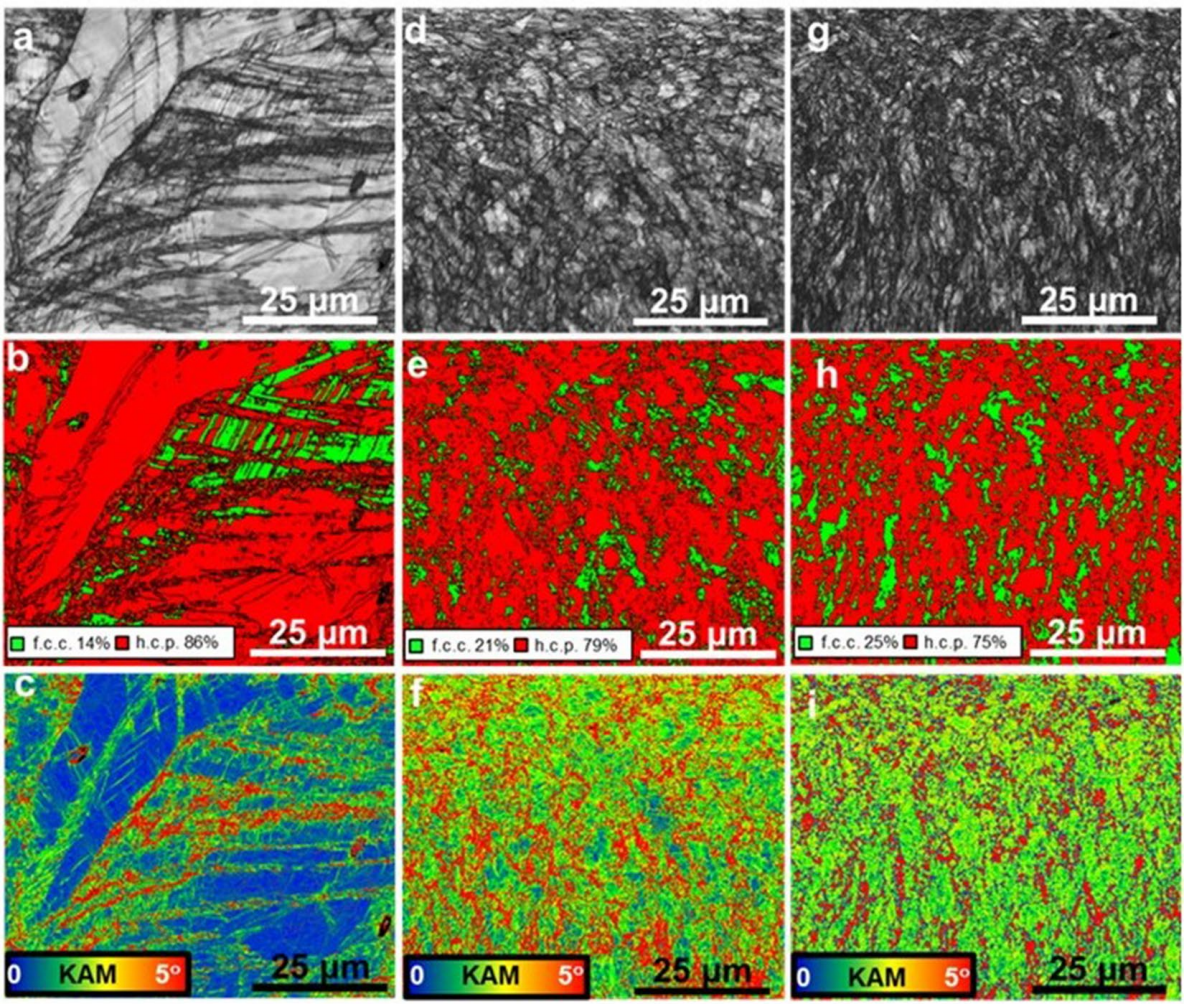
(**a**–**c**) EBSD image quality, phase, and KAM maps for the as-homogenized reference sample (**d**–**f**) image quality, phase, and KAM maps for the 350 RPM processed sample (**g**–**i**) image quality, phase, and KAM maps for the 650 RPM processed sample after room temperature tensile deformation at a strain rate of 10^−3^ s^−1^. RPM: rotations per minute; KAM: kernel average misorientation.

Figure 3a is an image quality map for the coarse-grained as-homogenized sample after tensile deformation, showing a heavily deformed microstructure. No significant changes in phase fraction were expected for this sample after deformation (Fig. 3b), as the starting volume fraction of f.c.c. γ-phase was extremely low. Most of the work hardening in this condition was associated with a dislocation density increase. KAM values were higher near the f.c.c. γ-phase, but quite low in the ε-phase region (Fig. 3c), indicating that most of the deformation was accommodated by the f.c.c. γ-phase. Thus for the as-homogenized material, dislocation assisted plasticity is the primary mechanism of deformation and not the TRIP effect.

These trends change drastically for FSP samples which showed very large deformation-induced transformation of f.c.c. γ- to h.c.p. ε-phase (Fig. 3e,h). Figure 3d,g are the associated image quality maps. Since the KAM values were above 1 in both, the f.c.c. γ- and the h.c.p. ε-phase after deformation for both FSP samples (Fig. 3e,f and h,i), they were directly associated with the densities of geometrically-necessary dislocations (GNDs)^13,14^. Regions with higher KAM values (indicated by red color) in Fig. 3c,f,i indicate larger plastic accommodation strains due to the strain mismatch among the two phases. As the deformation continues, the remaining f.c.c. γ-phase experiences gradually a higher barrier to the f.c.c. γ → h.c.p. ε transformation due to the back stresses exerted by the transformed h.c.p. ε-phase^3,4^.

Li *et al*.^3,4^ observed that at higher strain levels, the h.c.p. ε-phase also showed higher KAM values, suggesting that it accommodated some of the total deformation. Deformation-induced twinning is the primary mechanism by which the h.c.p. phase contributes to work hardening of the alloy^15–23^. Thus, the need for strain partitioning builds up during continuing deformation as the harder h.c.p. ε-phase volume fraction increases. FSP specimens before tensile deformation have lower average KAM values (Fig. 1e) than after tensile deformation (Fig. 3f,i), a circumstance that is likely to be related to lower GND arrays in the f.c.c. γ-phase. The fine initial γ grain size in the FSP processed specimens leads to a more homogeneous KAM pattern. The difference in KAM distribution (Fig. 3c,f,i) among the three conditions emphasizes the important role that microstructural features, such as the grain size and phase fractions, play for the deformation mechanisms in a TRIP HEA, as reported previously^3,4^.

### Enhanced strength-ductility index (SDI) for TRIP HEA: Effect of f.c.c. γ grain size and h.c.p. ε-phase fraction

**Figure 4:**
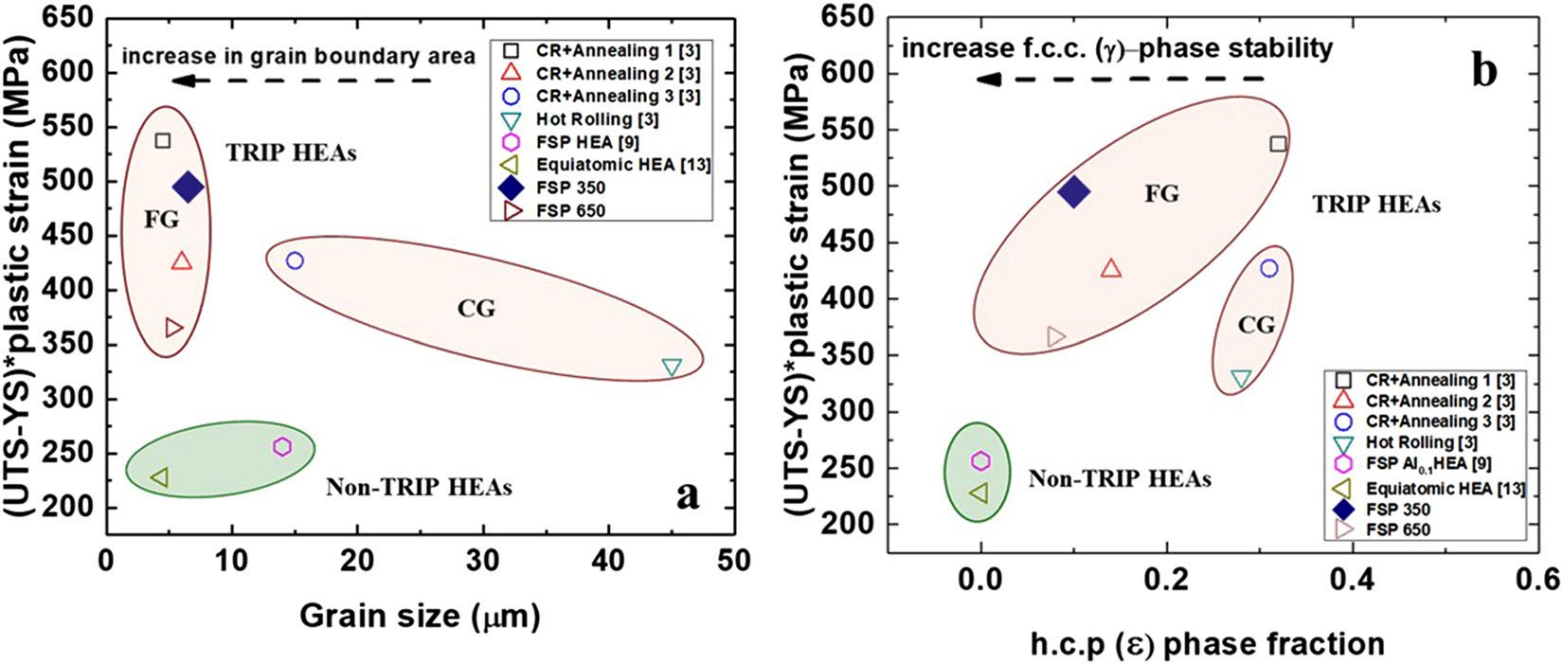
(**a**) Strength-ductility index (SDI) as a function of grain size and (**b**) variation of SDI as a function of ε phase fraction (prior to deformation) for grain-refined TRIP HEAs and non-TRIP HEAs. CG: coarse grained; FG: fine grained; FSP: friction stir processing; CR: cold rolling; RPM: rotations per minute.

Recent literature^15–23^ on TRIP steel, and work by Li *et al*.^3,4^ on TRIP HEAs have confirmed that the f.c.c. γ grain size and the h.c.p. ε-phase proportion are directly related to the thermodynamic stability of the f.c.c. γ-phase. Figure 4a,b show a strength-ductility index (SDI), defined as (UTS-YS) * plastic strain^24^, plotted as a function of grain size and h.c.p. ε-phase fraction for the TRIP HEA processed upon various processing conditions. The plot in Fig. 4a suggests that grain refinement in the TRIP HEAs leads to greater improvement in the SDI value than observed for the Fe_20_Mn_20_Co_20_Cr_20_Ni_20_ equiatomic reference HEA^25^. Moreover, a non-TRIP Al_0.1_CoCrFeNi HEA, after subjecting it to a FSP, showed a lower SDI value compared to TRIP HEAs with and without FSP exposure. This clearly indicates that TRIP HEAs have superior work hardenability when compared to more stable single phase HEAs, and have a strong potential to overcome the inverse relationship between strength and ductility.

Among the TRIP HEAs, it was apparent from Fig. 4b that SDI values are a function of f.c.c. γ grain size and h.c.p. ε-phase fraction prior to tensile deformation. Cold-rolled and annealed TRIP HEA (3 min at a furnace temperature of 900 °C followed by water quenching) showed a maximum SDI value^3,4^. Further, the material in FSP 350 RPM condition showed the second highest SDI value (with similar grain size) among all other TRIP HEAs treated by thermomechanical process, which could be attributed to the enhanced stability of the f.c.c. γ-phase after FSP. However, the difference in the SDI values for the 350 and 650 RPM treated specimens could be explained by considering both the grain refinement and the ε-phase fraction prior to tensile deformation.

The effect of grain size on f.c.c. γ-phase stability can be explained as follows: An increase in the grain boundary area per unit volume increases the number of preferential nucleation sites for h.c.p. ε-phase formation due to grain refinement. On the other hand, the adjacent grain boundaries restrict the growth of the h.c.p. ε plate by exerting back stresses and hence stabilize the f.c.c. γ grains. For example, the 350 RPM treated sample with an average grain size of 6.5 µm resulted in stabilization of the f.c.c. γ-phase as confirmed by the EBSD and XRD maps (Fig. 1c,d), respectively. The increase in strength, ductility and resultant SDI value of the alloy is tied to the refinement of grain size which leads to higher yield strength and larger number of nucleation sites for h.c.p. ε-phase resulting in improved work hardening.

Along with the finer grain size, the initial h.c.p. ε-phase fraction prior to tensile deformation also changes the overall stress-strain response of the metastable HEAs because of the change in the f.c.c. γ-phase stability and associated work hardenability. Therefore, relatively higher fractions of h.c.p. ε-phase with finer grain sizes has been suggested as a beneficial microstructure for metastable alloys for attaining substantial work hardening for improved strength and ductility^3,4,8^. Similar observations can be made from the present results, wherein the 350 RPM treated sample showed higher SDI value than the 650 RPM treated sample due to finer grain size and a higher fraction of the h.c.p. ε-phase prior to deformation. Alloys treated by conventional thermomechanical processes also displayed similar trends for the dependence of the SDI values on the h.c.p. ε-phase fractions. For example, the highest value of SDI was observed for a microstructure with finest grain size (4.5 µm) and almost 30% ε phase among the alloys and processing conditions considered in Fig. 4b ^3,4^.

The FSP engineered DP-HEA has a similar f.c.c γ average grain size (6.5 µm) as compared to the TMP-HEA reported by Li *et al*.^3,4^ (4.5 µm). However, the h.c.p ε-phase fraction of ~10% in the FSP engineered DP-HEA is lower than the value of 30% in TMP-HEA^3,4^. This difference in the starting h.c.p ε-phase fraction results in ~50 MPa difference in their SDI values, that of the TMP-HEA being higher. This comparison shows the intrinsic importance of not only grain size but also prior h.c.p. ε-phase fraction in optimizing the mechanical response of the DP-HEA irrespective of the processing path. Figure 4b reveal a processing window such that strength and ductility can be improved further by engineering both, the grain sizes and ε phase fractions to obtain excellent mechanical properties of TRIP HEAs.

## Conclusions

Friction stir processing resulted in a fine grain size along with a high volume fraction of f.c.c. (γ) phase as compared to as-homogenized reference material. This combination of fine grain size and distribution of small volume fraction of h.c.p. ε-phase leads to high work hardening rate over an extended plastic strain range due to an enhanced TRIP effect. The TRIP HEAs exhibit very high values of the strength-ductility product index and the response can be tailored through microstructural engineering. The initial FSP results also provide a first overview of the alloy’s suitability for joining via friction stir welding.

## Methods

### Materials and Processing

The TRIP HEA was produced by melting and casting in a vacuum induction furnace using pure metals with a nominal composition of Fe_50_Mn_30_Co_10_Cr_10_ (at %). The as-cast blocks were hot-rolled at 900 °C to a thickness of 50% (from 40 to 20 mm). Subsequently, the alloy sheets of 20 mm thickness were homogenized at 1200 C for 5 h in Ar atmosphere followed by quenching in ice water. Subsequently, sheets of 5 mm were machined out of the block by electro-discharge machining, and subjected to friction stir processing with the parameters mentioned in Table 1. The processing tool had a shoulder diameter of 12 mm with tapered pin. The root diameter, pin diameter, and length were 7.5 mm, 6 mm, and 3.5 mm, respectively.

**Table 1:** Processing parameters selected for FSP.

Processing parameters	
Rotational Rate (RPM)	350, 650
Traverse Speed (mm/min)	50.8
Plunge Depth (mm)	3.65
Tilt Angle (°)	2.5

### Microstructural and mechanical characterization

Microstructure of the alloy in homogenized (coarse-grained) and recrystallized (grain-refined) conditions were analyzed by various methods. X-ray diffraction (XRD) measurements were performed using an RIGAKU X-Ray equipment equipped with Cu K_α_ radiation operated at 40 kV and 30 mA. Electron backscatter diffraction (EBSD) measurements were carried out by a FEI NOVA Nano (SEM) with a Hikari camera and the TSL OIM data collection software.

Rectangular 1 mm-thick, dog-bone-shaped mini-tensile specimens were machined using a mini computer numerical control (CNC) machine from 1 mm below the top surface within the nugget region. Gage length and width of the tensile specimens were 5 and 1.25 mm, respectively. In each condition, three samples were tested at room temperature using initial strain rate of 10^−3^ s^−1^.
